# Milk as Food

**Published:** 1883-12

**Authors:** 


### MILK AS FOOD.

Unadulterated, undiluted, unskimmed, and properly treated milk, taken from a healthy cow in a good condition, and produced by the consumption of healthy and nutritious grasses and other kinds of food, contains within itself, in proper proportions, all the elements that are necessary to sustain human life through a considerable period of time. Scarcely any other single article of food will do this. When we eat bread and drink milk we eat bread, butter and cheese, and drink water, all of them in the best combination and condition to nourish the human system. All things considered, good milk is the cheapest kind of food that we have ; for 3 pints of it, weighing 3¾ pounds, and costing 9 cents, contain as much nutriment as one pound of beef, which costs 18 cents. There is no loss in cooking the milk, as there is in cooking beef, and there is no bone in it that cannot be eaten ; it is simple, palatable, nutritious healthful, cheap, and always ready for use, with or without preparation. This is to say that, chemically, 3.7 pounds of milk is the equivalent of 1 pound of beef in flesh-forming or nitrogenous constituents ; and 3.17 pounds of milk is the equivalent of 1 pound of beef in heat-producing elements or carbo-hydrates. We must therefore assume, from the data offered, that the relative values of beef and milk as human food are as 3½ to 11½, or as (in round numbers) 1 to 3⅓. If milk is 8 cents per quart, then it is the equal in food value to beef at 12½ cents per pound ; and *vice versa,* when beef is at 25 cents per pound, then milk should be 16 cents per quart, calculated on its food value. We thus see that, at any ruling prices, milk is certainly one of the cheapest, if not the cheapest food that can be furnished to the family, while all experience is in favor of its healthy qualities.—*Journal of Chemistry.*

